# Depression, alcohol use, and intimate partner violence among outpatients in rural Uganda: vulnerabilities for HIV, STIs and high risk sexual behavior

**DOI:** 10.1186/s12879-016-2162-2

**Published:** 2017-01-19

**Authors:** Susan M. Kiene, Haruna Lule, Katelyn M. Sileo, Kazi Priyanka Silmi, Rhoda K. Wanyenze

**Affiliations:** 1 Division of Epidemiology and Biostatistics, Graduate School of Public Health, San Diego State University, 5500 Campanile Drive (MC-4162), San Diego, CA 92182 USA; 2 Gombe General Hospital, Gombe, Uganda; 3 Department of Disease Control and Environmental Health, Makerere University School of Public Health, Kampala, Uganda

## Abstract

**Background:**

Intimate partner violence (IPV), alcohol use, and depression are key vulnerabilities for HIV in Uganda, and taken together may have a synergistic effect on risk. Our objective was to investigate the associations between depression, IPV, and alcohol use and HIV-risk indicators among a sample of outpatients in rural Uganda, and the effect of co-occurrence of these factors on HIV-risk indicators.

**Methods:**

In a structured interview we collected data on high-risk sexual behavior, depression symptoms, emotional and physical IPV, and alcohol use, as well as a blood sample for HIV and syphilis tests and a urine sample for chlamydia and gonorrhea tests from 325 male and female outpatients receiving provider-initiated HIV testing and counseling (PITC) at a public hospital outpatient clinic in rural Uganda. We used logistic regression and generalized linear modeling to test independent associations between depression, IPV, and alcohol use and HIV-risk indicators, as well as the effect of co-occurrence on HIV-risk indicators.

**Results:**

Twelve percent of men and 15% of women had two or more of the following conditions: depression, IPV, and alcohol use; another 29% of men and 33% of women had 1 condition. Each condition was independently associated with HIV risk behavior for men and women, and for women, depression was associated with testing positive for HIV or a sexually transmitted infection (STI). Men with one condition (AOR 2.32, 95% CI 1.95–2.77) and two or more conditions (AOR 12.77, 95% CI 7.97–20.47) reported more high risk sex acts compared to those with no potential co-occurring conditions. For men, experiencing two or more conditions increased risky sex more than one alone (*χ* ^2^ 24.68, *p* < 0.001). Women experiencing one condition (AOR 3.33, 95% CI 137–8.08) and two co-occurring conditions (AOR 5.87, 95% CI 1.99–17.35) were more likely to test positive for HIV or an STI and women with two co-occurring conditions were also at increased risk for risky sex (AOR 2.18, 95% CI 1.64–2.91). We also found preliminary evidence suggesting synergistic effects between depression and emotional IPV and between alcohol use and depression.

**Conclusions:**

This study demonstrates the co-occurrence of depression, IPV, and alcohol use in men and women in an outpatient setting in rural Uganda. The co-occurrence of these factors was associated with greater HIV risk, highlighting the need for a more holistic approach to HIV prevention and care research and programming.

## Background

Although there has been an overall 33% decrease of HIV incidence in sub-Saharan Africa, Uganda is one of two countries that recorded an increase in new HIV infections between the period of 2005 and 2013 [1]. The national prevalence for 2012–2013 was estimated at 7.4% among the adult population and a total of 1.5 million people were estimated to be living with HIV in Uganda in 2014 [2]. Despite governmental efforts, the 2014 HIV incidence of 95,000 was substantially behind the target of 71,510 [2]. As efforts to end the HIV epidemic accelerate, more research is needed to identify those most vulnerable to acquiring HIV and those at highest risk of transmitting HIV [3]. While focus on key populations at highest risk such as fishermen and sex workers in Uganda is paramount [2, 4], the majority of new infections are among those not within a key population [2]. Thus, there remains a need for a deeper understanding of risk factors for HIV infection in the general population in Uganda.

Research throughout sub-Saharan Africa points to intimate partner violence (IPV), alcohol consumption, and depression as vulnerabilities for HIV [5–10]. In Uganda, all three conditions are prevalent and each have been linked in the literature to HIV risk. IPV for example is associated with risk of HIV infection, alcohol consumption of the male partner, multiple sexual partners, inconsistent/non-use of condoms and sexual coercion [9, 10]. IPV is particularly high in Uganda, with 27% of women reporting experiencing physical IPV in the prior 12 months [11]. Alcohol use, which is also high in Uganda, is similarly associated with low or inconsistent condom use, greater number of sex partners, and more extramarital sex [5–8], as well as HIV risk behavior across settings [12–15]. Finally, the link between depression and HIV risk has been established in the broader literature [16]. In Uganda, most research on depression and HIV focus on people who are already HIV infected, and report an association of depression with lower condom use, higher alcohol use, lower self-efficacy, lower CD4 count, and poorer antiretroviral treatment adherence [17–20]. One study that examined the association of mental health with HIV risk behavior in Ugandan university students found depression was significantly related to an increased number of sexual partners among both males and females [21]. Similar associations have been found in other sub-Saharan African settings, linking depression to HIV risk behavior [22–24].

Global agencies and public health experts advocate for “combination prevention” strategies that address biomedical, behavioral and social/structural prevention strategies and operate on multiple ecological levels [25–27]. Such a holistic approach requires understanding of co-occurrence of individual conditions like alcohol consumption and depression along with societal determinants of health such as IPV that increase HIV risk behavior and HIV acquisition. While studies from Uganda and sub-Saharan Africa have identified individual or dual associations of risky sexual behavior with high alcohol consumption, IPV and depression [5–8, 18, 28–32], few studies have looked into the effect of the co-occurrence of all three of these factors on HIV risk behaviors in sub-Saharan Africa [33]. The global literature focusing on *syndemics*, or the co-occurrence of multiple diseases or conditions which exacerbate the effect of the conditions on health, suggests that the multiple pathways that link HIV risk with IPV, alcohol use, and depression are complex and multidimensional [34, 35]. Thus, there is a need to understand how IPV, alcohol consumption and depression intersect and together compound HIV risk. The objective of the present study was to investigate the associations between depression, IPV, and alcohol use and HIV-risk indicators among a sample of outpatients in rural Uganda. Outcomes examined include: high risk sex and testing positive for HIV or an STI (syphilis, gonococcal urethritis, chlamydial urethritis). In addition, we investigated the co-occurrence of depression, IPV, and alcohol use and potential effects of their co-occurrence and interaction on our HIV-risk-related outcomes.

## Methods

### Setting, recruitment, and eligibility criteria

This cross-sectional study uses baseline data from a study testing a brief HIV risk reduction intervention implemented during provider-initiated HIV testing and counseling (PITC) at a public hospital outpatient clinic in rural Uganda [36]. At this hospital, and most hospitals in Uganda, PITC is routinely offered to all patients attending the outpatient clinic regardless of the reason for the visit. A research assistant non-systematically approached and recruited individuals who were waiting in the outpatient waiting area to be seen by a clinician. Further details about recruitment can be found in a previous publication from the parent study [36]. The research assistant briefly described the study to potential participants, determined eligibility, and obtained written informed consent from those eligible and interested in participating. Eligibility criteria were: 18 years of age or older, residing within 20 km of the hospital, not having tested for HIV within the prior 6 months, sexually active in the prior month, not attending the clinic specifically for HIV testing, not having previously tested positive for HIV, and not pregnant. We purposely recruited approximately equal numbers of men and women.

### Procedures

Participants completed a structured interviewer-administered computer-assisted personal interview (CAPI) in Luganda in the onsite study office. Rapid HIV testing during PITC was performed according to the Ugandan Ministry of Health protocol [37]. Determine HIV-1/2 Assay test kits were used. Those found reactive with Determine were immediately confirmed reactive using HIV 1/2 STAT-PAK. If reactive with STAT-PAK, they were confirmed positive. Uni-Gold HIV was used as a tie breaker if the patient was found reactive using Determine but nonreactive to STAT-PAK. Participants also provided a first catch urine sample for *chlamydia trachomatis* (CT) and *neisseria gonorrhoeae* (NG) testing using Roche Amplicor polymerase chain reaction (PCR). Remnant plasma from the HIV test specimen was used to test for Syphilis using Rapid plasma reagin (RPR) with confirmation by *Treponema pallidum* hemagglutination (TPHA).

### Measures

The baseline questionnaire included participant sociodemographics, detailed questions about their sexual behavior during the prior 3 months, as well as questions measuring alcohol use, depression symptoms, and experience of IPV. We used timeline follow back techniques [38] to help participants recall and report details about their sexual behavior during the prior 3 months including: (1) the number of sexual partners, (2) knowledge of each partner’s HIV testing history and test results, (3) the number of vaginal and anal sex acts with each partner, and (4) how many of the reported sex acts with each partner were protected by a condom. *High-risk sex acts* were considered to be unprotected sex acts with a partner of unknown or HIV positive status—representing potential risk from a participant’s perspective. Alcohol use was assessed using the 10 question AUDIT (Alcohol Use Disorders Identification Test) which is designed to identify potentially problematic drinking [39]. Alcohol use was categorized into three categories: no drinking, low risk drinking (AUDIT score of 1–6 for women, 1–7 for men), and potentially problematic (harmful/hazardous) drinking (AUDIT score ≥7 for women, ≥8 for men). To assess depression symptoms we used the short version of the Hopkins Symptom Checklist [40] which contains 15 items on a 1 (not at all) to 4 (extremely) scale. This measure has been validated in East African settings [41]. The scores on all items are summed and then a mean calculated with a mean score of 1.75 or above indicating possible depression [42–44]. Experience of IPV perpetrated by their current partner within the prior 12 months was assessed using a measure adapted from the WHO violence against women instrument [45] to be applicable to both genders. We used the items from the instrument to ask about experiences of emotional abuse, 4 items (e.g., *Has he/she insulted you or made you feel bad about yourself?*) and physical violence, six items (e.g., *Has he/she slapped you or thrown something at you that could hurt you?*). For our biological measures, a person was categorized as having an STI or HIV if he/she tested positive for one or more of the following: HIV, syphilis, chlamydia, or gonorrhea. These were combined into one variable since, in this Ugandan context, HIV and these STIs have largely the same risk factors. Moreover, the cell sizes for individual pathogens or HIV alone and STIs combined would limit our statistical power to detect associations of interest.

### Statistical analysis

We used logistic regression for binary outcome variables. For the outcome of number of high risk sex acts, we modeled these as events within trials, with events being the number of high risk vaginal sex acts and trials being the number of vaginal sex acts, using generalized linear modeling with a binomial distribution and logit link. This models high risk sex acts as the outcome relative to a participant’s total number of sex acts. We conducted all analyses with SPSS version 24 [46] and ran models separately for men and women since we expected that the experience of IPV in particular is different for men and women.

We first examined bivariate relationships between sociodemographic factors and depression, IPV, alcohol use, and high risk sex and testing positive for HIV, syphilis, chlamydia, or gonorrhea to determine which to control for in subsequent analyses. Sociodemographic variables associated with any of the key variables at *p* < 0.25 were included in subsequent analyses. Next, in a multivariable model including all of the sociodemographic factors and each of the potential co-occurring conditions, we explored the associations between each of the conditions and the outcomes of high risk sex and testing positive for HIV or an STI. To examine co-occurrence of conditions we tested all bivariate associations between depression, alcohol use, and IPV controlling for sociodemographic variables. We also explored if having more co-occurring conditions was associated with greater HIV-risk by summing each participant’s number of co-occurring conditions and tested this as a categorical predictor of behavioral and biological HIV risk indicators controlling for sociodemographic factors. Because of small cell sizes, we used three categories: 0, 1, and 2 or more co-occurring conditions. Finally, in exploratory analyses, we tested all two-way interactions between the co-occurring conditions of depression, IPV, and alcohol use by adding these interactions to the multivariate model to examine potential synergistic effects of these syndemic conditions. We dichotomized alcohol use into yes/no to make the testing of interactions feasible in this model. We did not test three-way interactions due to small cell sizes.

## Results

A total of 325 outpatients (160 men, 165 women) receiving PITC were included in the analytic sample. From the full sample reported on elsewhere [36], we excluded five men and three women because they had inconclusive results on the STI tests. We report participant characteristics in Table 1 including sociodemographics. Men reported an average of 22.70 (SD 18.07) and women reported an average of 16.42 (SD 11.97) high risk vaginal sex acts in the prior 3 months. No participants reported anal sex. Twenty men (12.5%) and 38 women (23.0%) tested positive for one or more of the following: HIV, syphilis, chlamydia, or gonorrhea. For HIV, 6.9% of men and 12.7% of women tested positive; 1.9% of men and 8.7% of women tested positive for syphilis; 1.9% of men and 2.5% of women tested positive for chlamydia; and 1.9% of men and 3.7% of women tested positive for gonorrhea.

**Table 1:** Sample characteristics

	Males (*n* = 160) Number (%) Mean (SD)	Females (*n* = 165) Number (%) Mean (SD)
Age	34.93 (10.59)	32.21 (8.93)
Marital status		
Married	132 (82.5%)	149 (90.3%)
Unmarried	28 (17.5%)	16 (9.7%)
Education		
Secondary >	71 (44.4%)	48 (29.1%)
Primary	84 (52.5%)	106 (64.2%)
No formal education	5 (3.1%)	11 (6.7%)
Employment		
Throughout the year	86 (53.8%)	40 (24.2%)
Part of the year	36 (22.5%)	34 (20.6%)
Once in a while/Never	38 (23.7%)	91 (55.2%)
Depression		
Yes	23 (14.4%)	40 (24.2%)
No	137 (85.6%)	125 (75.8%)
Emotional intimate partner violence		
Yes	32 (20.0%)	38 (23.0%)
No	128 (80.0%)	127 (77.0%)
Physical intimate partner violence		
Yes	9 (5.6%)	27 (16.4%)
No	151 (94.4%)	138 (83.6%)
Alcohol use (AUDIT)		
Problematic drinking^a^	23 (14.4%)	8 (4.8%)
Low risk drinking	10 (6.3%)	9 (5.5%)
No drinking	127 (79.4%)	148 (89.7%)
Number of risky sex acts in prior 3 months	22.70 (18.07)	16.42 (11.97)
Tested positive for HIV or an STI		
Yes	19 (12.5%)	38 (23.0%)
No	140 (87.5%)	127 (77.0%)
Number of syndemic conditions^b^		
3	4 (2.5%)	0 (0.0%)
2	15 (9.4%)	24 (14.5%)
1	46 (28.8%)	54 (32.7%)

^a^AUDIT classification of problematic drinking is a score ≥ 7 for females, ≥ 8 for males

^b^If participant reported both emotional and physical IPV it is counted as only one syndemic condition

A quarter of women (24.2%) and 14.4% of men screened positive for possible depression. Approximately 14% of men and 5% of women had AUDIT scores indicating problematic drinking, 6.3% of men and 5.5% of women had scores indicating low risk drinking, and 79.4% of men and 89.7% of women reported no alcohol use in the prior year. Experience of IPV perpetrated by their current partner in the prior 12 months was prevalent among both women and men with 23.0% of women and 20.0% of men reporting emotional IPV and 16.4% of women and 5.6% of men reporting physical IPV. Physical IPV in the absence of emotional IPV was rare (4.2% of women, 0% of men). See Table 1 for additional details about the sample characteristics.

In Tables 2 and 3 we report the results of our bivariate and multivariate models testing the associations between demographic characteristics and co-occurring conditions and our outcomes of high-risk sex acts and testing positive for HIV or an STI. We report unadjusted results from bivariate models and adjusted results from the multivariate model. In the multivariate model, estimates are adjusted for all variables included in the model. The multivariate model showed that among men, more education, more frequent employment, depression, emotional IPV, physical IPV, problematic drinking and lower risk drinking were all statistically significant predictors of greater numbers of high risk sex acts (see Table 2). Marital status and age were the only variables not associated with high risk sex acts among men. Among women, greater age, being unmarried, more frequent employment, depression, emotional IPV, and lower risk drinking were associated with reporting more high risk sex acts. Physical IPV was associated with fewer high risk sex acts. Education was not associated with high risk sex among women. Results from the multivariate model examining predictors of a positive HIV or STI test (see Table 3) showed that among men, none of the demographic or potentially co-occurring conditions were significantly associated with testing positive for HIV or an STI. Women screening positive for depression were more likely to test positive for HIV or an STI. Among women, emotional IPV appeared to be associated with a positive HIV or STI test, although this did not reach conventional statistical significance (*p* < 0.05).

**Table 2:** Results from bivariate and multivariate regression models examining associations between the number or risky sex acts relative to the number of sex acts and sociodemographics and syndemic conditions

	Males (*n* = 160)				Females (*n* = 165)			
	Number of risky sex acts OR 95% CI	*χ*2 *p* value	Number of risky sex acts AOR 95% CI	*χ*2 *p* value	Number of risky sex acts OR 95% CI	*χ*2 *p* value	Number of risky sex acts AOR 95% CI	*χ*2 *p* value
Age	1.02 (1.01–1.03)	*χ* ^2^ = 24.83, *p* < 0.001	1.00 (0.99–1.01)	*χ* ^2^ = 0.20, *p* = 0.65	1.01 (0.99–1.02)	*χ* ^2^ = 3.24, *p* = 0.07	1.02 (1.01–1.03)	*χ* ^2^ = 9.36, *p* = 0.002
Marital status		*χ* ^2^ = 4.44, *p* = 0.04		*χ* ^2^ = 0.68, *p* = 0.41		*χ* ^2^ = 0.06, *p* = 0.80		*χ* ^2^ = 6.41, *p* = 0.01
Married	1.32 (1.02–1.70)		1.13 (0.85–1.52)		0.95 (0.66–1.38)		0.60 (0.40–0.89)	
Unmarried	-		-		-		-	
Education		*χ* ^2^ = 159.08, df = 2, *p* < 0.001		*χ* ^2^ = 124.99, df = 2, *p* <0.001		*χ* ^2^ = 4.89, df = 2, *p* = 0.09		*χ* ^2^ = 3.77, df = 2, *p* = 0.15
Secondary >	0.78 (0.51–1.18)		1.83 (1.10–3.03)		1.17 (0.83–1.63)		0.75 (0.53–1.08)	
Primary	1.92 (1.27–2.92)		4.24 (2.56–7.04)		1.35 (0.98–1.86)		0.89 (0.63–1.25)	
No formal education	-		-					
Employment		*χ* ^2^ = 46.30, df = 2, *p* < 0.001		*χ* ^2^ = 15.17, df = 2, *p* = 0.001		*χ* ^2^ = 57.00, df = 2, *p* < 0.001		*χ* ^2^ = 54.35, df = 2, *p* < 0.001
Throughout the year	1.03 (0.87–1.22)		1.37 (1.13–1.67)		2.30 (1.82–2.90)		2.32 (1.82–2.97)	
Part of the year	1.92 (1.54–2.39)		1.61 (1.25–2.07)		1.64 (1.30–2.06)		1.72 (1.36–2.19)	
Once in a while/never	-		-		-		-	
Depression		*χ* ^2^ = 99.64, *p* < 0.001		*χ* ^2^ = 68.27, *p* < 0.001		*χ* ^2^ = 22.32, *p* < 0.001		*χ* ^2^ = 22.20, *p* < 0.001
Yes	4.19 (3.16–5.56)		3.50 (2.60–4.71)		1.70 (1.37–2.12)		1.79 (1.40–2.28)	
No	-		-		-		-	
Emotional IPV		*χ* ^2^ = 87.69, *p* < 0.001		*χ* ^2^ = 4.55, *p* = 0.03		*χ* ^2^ = 14.42, *p* < 0.001		*χ* ^2^ = 26.17, *p* < 0.001
Yes	2.68 (2.18–2.93)		1.31 (1.02–1.67)		1.50 (1.22–1.85)		1.92 (1.50–2.47)	
No	-		-		-		-	
Physical IPV		*χ* ^2^ = 49.24, *p* < 0.001		*χ* ^2^ = 26.49, *p* < 0.001		*χ* ^2^ = 0.03, *p* = 0.87		*χ* ^2^ = 6.89, *p* = 0.009
Yes	6.78 (3.93–11.69)		5.23 (2.78–9.81)		0.98 (0.79–1.22)		0.69 (0.53–0.91)	
No	-		-		-		-	
Alcohol use (AUDIT)		*χ* ^2^ = 117.48, df = 2, *p* < 0.001		*χ* ^2^ = 15.61, df = 2, *p* < 0.001		*χ* ^2^ = 18.58, df = 2, *p* < 0.001		*χ* ^2^ = 15.61, df = 2, *p* < 0.001
Problematic drinking^a^	2.48 (1.93–3.18)		2.65 (2.00–3.50)		1.00 (0.62–1.61)		0.75 (0.45–1.25)	
Lower risk drinking	8.49 (5.18–13.93)		9.10 (5.46–15.16)		3.56 (2.00–6.33)		3.14 (1.73–5.67)	
No drinking	-		-		-		-	

Note: OR = Odds Ratio; AOR = Adjusted Odds Ratio; 95% CI = 95% Confidence Interval

^a^ AUDIT classification of hazardous/harmful drinking is a score ≥ 7 for females, ≥ 8 for males

**Table 3:** Results from bivariate and multivariate regression models examining associations between testing positive for HIV or STI and sociodemographics and syndemic conditions

	Males (*n* = 160)				Females (*n* = 165)			
	HIV or STI OR 95% CI	*χ*2 *p* value	HIV or STI AOR 95% CI	*χ*2 *p* value	HIV or STI OR 95% CI	*χ*2 *p* value	HIV or STI AOR 95% CI	*χ*2 *p* value
Age	0.98 (0.95–1.02)	*χ* ^2^ = 1.62, *p* = 0.20	0.99 (0.94–1.04)	*χ* ^2^ = 0.29, *p* = 0.59	1.03 (0.99–1.07)	*χ* ^2^ = 1.84, *p* = 0.18	1.05 (1.00–1.10)	*χ* ^2^ = 3.37, *p* = 0.066
Marital status		*χ* ^2^ = 0.82, *p* = 0.37				*χ* ^2^ = 0.19, *p* = 0.66		
Married	2.02 (0.44–9.24)				1.32 (0.36–4.98)			
Unmarried	-				-			
Education		*χ* ^2^ = 3.24, df = 2, *p* = 0.20		*χ* ^2^ = 1.57, df = 2, *p* = 0.46		*χ* ^2^ = 0.32, df = 2, *p* = 0.85		
Secondary >	0.17 (0.02–1.17)		0.27 (0.03–2.19)		1.54 (0.29–8.17)			
Primary	0.22 (0.03–1.45)		0.30 (0.04–2.25)		1.32 (0.27–6.51)			
No formal education	-		-					
Employment		*χ* ^2^ = 3.37, df = 2, *p* = 0.19		*χ* ^2^ = 1.64, df = 2, *p* = 0.44		*χ* ^2^ = 0.95, df = 2, *p* = 0.62		
Throughout the year	0.84 (0.24–2.97)		1.06 (0.28–4.04)		1.45 (0.61–3.43)			
Part of the year	2.20 (0.60–8.06)		2.07 (0.53–8.12)		1.42 (0.57–3.57)			
Once in a while/never	-		-		-			
Depression		*χ* ^2^ = 2.09, *p* = 0.15		*χ* ^2^ = 0.94, *p* = 0.33		*χ* ^2^ = 10.39, *p* = 0.001		*χ* ^2^ = 8.18, *p* = 0.004
Yes	2.30 (0.74–7.08)		1.86 (0.53–6.52)		3.63 (1.66–7.93)		3.42 (1.48–7.92)	
No	-		-		-		-	
Emotional IPV		*χ* ^2^ = 6.69, *p* = 0.01		*χ* ^2^ = 0.13, *p* = 0.72		*χ* ^2^ = 3.01, *p* = 0.08		*χ* ^2^ = 2.73, *p* = 0.09
Yes	1.92 (1.02–2.42)		1.30 (0.32–5.30)		2.07 (0.91–4.69)		2.53 (0.84–7.60)	
No	-		-		-		-	
Physical IPV		*χ* ^2^ = 2.03, *p* = 0.15		*χ* ^2^ = 0.11, *p* = 0.74		*χ* ^2^ = 1.38, *p* = 0.24		*χ* ^2^ = 0.12, *p* = 0.73
Yes	2.14 (0.07–7.37)		1.41 (0.18–10.87)		1.60 (0.72–3.77)		0.81 (0.24–2.68)	
No	-		-		-		-	
Alcohol use (AUDIT)		*χ* ^2^ = 2.82, df = 2, *p* = 0.24		*χ* ^2^ = 0.47, df = 2, *p* = 0.79		*χ* ^2^ = 4.12, df = 2, *p* = 0.13		*χ* ^2^ = 3.63, df = 2, *p* = 0.16
Problematic drinking^a^	1.70 (0.83–3.51)		1.56 (0.37–6.57)		2.25 (0.90–4.92)		1.53 (0.30–7.81)	
Lower risk drinking	0.85 (0.35–2.24)		0.78 (0.08–7.46)		2.99 (0.85–8.93)		4.27 (0.93–19.71)	
No drinking	-		-		-		-	

Note: OR = Odds Ratio; AOR = Adjusted Odds Ratio; 95% CI = 95% Confidence Interval

^a^ AUDIT classification of problematic drinking is a score ≥ 7 for females, ≥ 8 for males

Table 4 presents the bivariate associations between the potential co-occurring conditions. Among men, the associations between: depression and emotional IPV; problematic alcohol use and emotional IPV; lower risk drinking and emotional IPV; and lower risk drinking and physical IPV were statistically significant at *p* < 0.05. Among women, only the associations between depression and emotional IPV and depression and physical IPV were statistically significant. Based on these results, alcohol use, as one of the potential co-occurring conditions, was excluded from subsequent analyses among women, since it did not correlate with the other conditions and was low prevalence among women.

**Table 4:** Correlations between syndemic conditions for males and females

Syndemic condition	Depression		Problematic drinking Low risk drinking	
	OR (95% CI)	*p*	OR (95% CI)	*p*
Males (*N*=160)				
Depression	-	-	1.33 (0.41-4.37)	0.64
			0.70 (0.08-5.89)	0.75
Emotional IPV	3.33 (1.29-8.59)	0.013	3.82 (1.45-10.07)	0.007
			3.97 (1.02-15.38)	0.046
Physical IPV	1.84 (0.36-9.44)	0.47	2.42 (0.44-13.29)	0.31
			6.35 (1.06-37.98)	0.04
Females (*N*=165)				
Depression	-	-	2.07 (0.47-9.08)	0.34
			1.72 (0.41-7.24)	0.46
Emotional IPV	2.33 (1.06-5.13)	0.035	2.07 (0.47-9.08)	0.34
			0.43 (0.05-3.56)	0.43
Physical IPV	3.23 (1.36-7.66)	0.008	1.76 (0.34-9.27)	0.50
			0.66 (0.08-5.53)	0.70

*Note*: *OR* Odds Ratio; *95% CI* 95% Confidence Interval; Correlations were run using logistic regression. Correlations between emotional and physical IPV cannot be calculated because physical IPV always co-occurred with emotional IPV. Correlations with alcohol use are separated by level of alcohol use (harmful/hazardous and low risk) according to the AUDIT risk level classifications

**Fig. 1:**
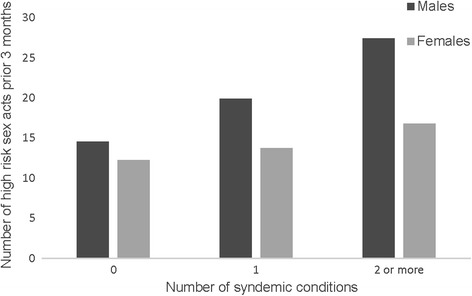
Predicted values of number of high risk sex acts in the prior 3 months, adjusting for all covariates (age, marital status, employment, education)

**Fig. 2:**
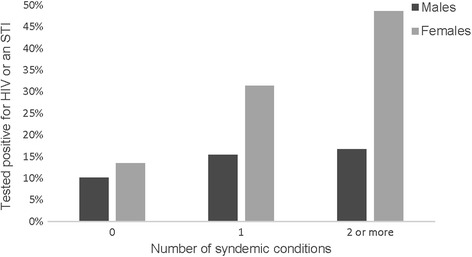
Predicted values of percentage tested positive for HIV or an STI, adjusting for all covariates (age, marital status, employment, education)

Approximately 40% of men (40.6%) and 47.3% of women reported one or more of the following conditions: depression, emotional or physical IPV, alcohol use. For the purpose of the next analysis the two types of IPV were counted as one syndemic condition and alcohol use was dichotomized into yes/no. As shown in Table 1, 28.8% of men and 32.7% of women had one, 9.4% of men and 14.5% of women had two, and 2.5% of men had all three conditions. The co-occurrence of two syndemic conditions was distributed as: 8 males and 4 females had alcohol use and IPV, 6 males and 15 females had depression and IPV, and 1 male and 5 females had alcohol use and depression. In examining how co-occurring conditions are associated with HIV-risk indicators (see Table 5), we found that among both men and women, compared to those with no potential co-occurring conditions, those with 2 or more conditions reported more high risk sex acts relative to their number of sex acts (Men: AOR 12.77, 95% CI 7.97–20.47, *χ* ^2^ 112.08, *p* < 0.001; Women: AOR 2.18, 95% CI 1.64–2.91, *χ* ^2^ 28.30, *p* < 0.001). Similarly, for men but not women, those experiencing one condition reported more high risk sex than did those experiencing none (AOR 2.32, 95% CI 1.95–2.77, *χ* ^2^ 87.40, *p* < 0.001). The pairwise comparison with Bonferroni correction between 2 or more vs 1 condition among men was also statistically significant (*χ* ^2^ 24.68, *p* < 0.001), showing that experiencing 2 or more conditions increased risk more than 1 alone. Figure 1 shows these relationships. As illustrated in Fig. 2, women experiencing 2 conditions had 5 times greater odds of testing positive for either HIV or an STI (AOR 5.87, 95% CI 1.99–17.35, *χ* ^2^ 10.23, *p* = 0.001), and those experiencing one condition had more than three times greater odds of testing positive for either HIV or an STI (AOR 3.33, 95% CI 1.37–8.08, *χ* ^2^ 7.08, *p* = 0.008) compared to those with no potentially co-occurring conditions. However, in pairwise comparisons having 2 or more conditions was not significantly different than 1 in terms of testing positive for HIV or an STI (*χ* ^2^ 3.15, *p* = 0.08). Among men there was no significant association between the number of potentially co-occurring conditions and the odds of testing positive for HIV or an STI.

**Table 5:** Co-occurring conditions and associations with high risk sex and testing positive for HIV or an STI

AOR		95% CI	*χ* ^2^	*p*
Males (*N* = 160)				
High risk sex acts				
2 or more conditions	12.77	7.97–20.47	112.08	<0.001
1 condition	2.32	1.95–2.77	87.40	<0.001
HIV/STI				
2 or more conditions	1.20	0.27–5.37	0.05	0.82
1 condition	1.82	0.61–5.41	1.14	0.29
Females (*N* = 165)				
High risk sex acts				
2 or more conditions	2.18	1.64–2.91	28.30	<0.001
1 condition	1.11	0.92–1.34	1.11	0.29
HIV/STI				
2 or more conditions	5.87	1.99–17.35	10.23	0.001
1 condition	3.33	1.37–8.08	7.08	0.008

Note: AOR = Adjusted Odds Ratio; 95% CI = 95% Confidence interval. Models adjusted for sociodemographic covariates

**Fig. 3:**
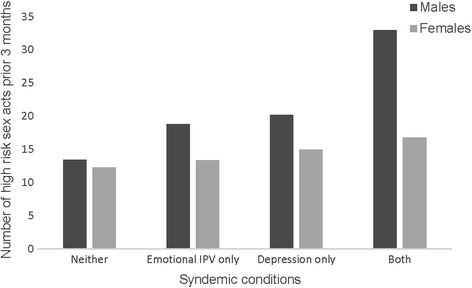
Synergistic effect of syndemic conditions. Predicted values of number of high risk sex acts in the prior 3 months, adjusting for all covariates (age, marital status, employment, education)

**Fig. 4:**
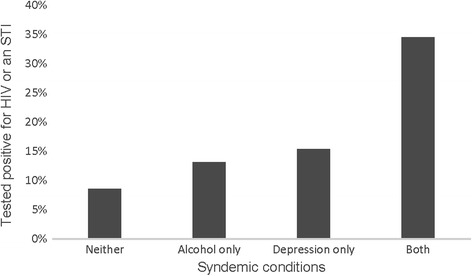
Synergistic effect of syndemic conditions for males. Predicted values of percentage tested positive for HIV or an STI, adjusting for all covariates (age, marital status, employment, education)

Table 6 presents the results from our exploratory analysis examining potential synergistic effects of 2 co-occurring syndemic conditions on risky sex acts and testing positive for HIV or an STI. Adding the interaction terms to our multivariate models from Tables 2 and 3, we found evidence of synergistic effects between depression and emotional IPV on high risk sex acts among both women and men (interaction AOR women: 2.05, 95% CI 1.52–4.00, *p* < 0.001; men: interaction AOR 13.15, 95% CI 1.77–97.61, *p* = 0.01). Figure 3 illustrates this synergistic effect. The effect was particularly strong for men; having either depression or experiencing emotional IPV alone was associated with more high risk sex acts but together they were associated with even greater high risk sex. This synergistic effect among women, while statistically significant, was much less pronounced. We also found evidence of a synergistic effect among men between depression and alcohol use on testing positive for HIV (interaction AOR 18.09, 95% CI 1.10–298.96, *p* = 0.04). This effect is illustrated in Fig. 4, which like the previous synergistic effect, shows large increases in the odds of testing positive for HIV or an STI for men having both depression and drinking alcohol compared to the smaller increases from either one of these conditions alone.

**Table 6:** Tests of interactions between syndemic conditions on high risk sex and testing positive for HIV or an STI

	High risk sex acts AOR, 95% CI, p	Tested positive for HIV or STI AOR, 95% CI, *p*
Males (*N* = 160)		
Depression x emotional IPV	13.15 (1.77–97.61), *p* = 0.01	2.25 (0.19–26.20), *p* = 0.52
Depression x alcohol	^a^	18.09 (1.10–298.96) *p* = 0.04
Alcohol x emotional IPV	1.67 (0.92–3.02), *p* = 0.09	1.52 (0.14–16.81), *p* = 0.73
Depression x physical IPV	0.36 (0.04–3.01), *p* = 0.35	2.99 (0.07–121.24), *p* = 0.56
Alcohol x physical IPV	^a^	0.97 (0.03–29.43), *p* = 0.97
Females (*N* = 165)		
Depression x emotional IPV	2.50 (1.52–4.00), *p* < 0.001	1.20 (0.20–7.15), *p* = 0.85
Depression x physical IPV	1.44 (0.87–2.38), *p* = 0.16	1.04 (0.14–7.94), *p* = 0.97

Note: AOR = Odds Ratio; 95% CI = 95% Confidence interval. Models adjusted for sociodemographic covariates

^a^ model cannot run due to low variability in the outcome due to small cell sizes. Interactions were tested as part of the full multivariate models reported in Tables 2 and 3 but only the interactions are reported in this table

## Discussion

This study is the first to our knowledge to assess the independent and co-occurring associations between depression, IPV, and alcohol use with both high risk sex and biomarkers of HIV and STIs among a sample of outpatients in rural Uganda. Depression, IPV, and alcohol use were co-occurring among men and women in our sample, with the exception of alcohol use for women. Among men and women, each of these factors were independently associated with high risk sexual behavior, and for women, depression was associated with testing positive for HIV or an STI. Taken together, experiencing multiple conditions was associated with a greater likelihood of engaging in high risk sex for men and women, and greater odds of testing positive for HIV or an STI for women. We also found some evidence of synergistic effects between depression and emotional IPV on high risk sex and between depression and alcohol on testing positive for HIV or an STI among men, although we had limited power to test these interactions.

Our findings add support for the co-occurrence of depression, IPV, and alcohol use [47–49]. In our sample, 12% of men and 15% of women reported two or more of these conditions. Alcohol use was overall low among both men and women, which may be explained by the rural setting and Muslim population (42%). However, over half of men and nearly half of women who drank were classified as having problematic alcohol use, reflecting potential harmful and hazardous drinking. Prior studies have shed light on how depression, IPV, and alcohol use may be interrelated; for example, experiencing IPV may result in depression and other mental health conditions [49–51], and those experiencing IPV and/or depression may engage in alcohol use as a coping mechanism [48]. These associations may also be bidirectional; depression, and one’s risk of experiencing and perpetrating IPV, may be exacerbated by alcohol use [48, 52]. Our findings similarly add to a growing literature that establishes each of these conditions as independent predictors of HIV risk behavior and HIV/STI acquisition [5–8, 18, 28–30]. Less research, however, has examined how men’s experiences of IPV affects their HIV risk, which in our study was associated with HIV risk behavior. Experience of emotional IPV among men was high, but comparable to that reported among women, as well as the rates reported among men in the Uganda National Demographic and Health Survey (33% of men report any lifetime emotional IPV from a spouse) [11]. The causes and consequences of experiencing emotional IPV among men have not been examined in rural Uganda. Given the cultural significance of men’s role as financial providers in this setting, future research could explore this as a possible source of spousal conflict and female-perpetrated IPV. As expected, experience of physical IPV among men in our sample was significantly lower than among women.

This study uniquely adds to the literature by demonstrating that the co-occurrence of depression, IPV, and alcohol use is associated with high risk sexual behavior and HIV/STI outcomes in Uganda. We also found some preliminary evidence of the potential synergistic effects of some of these conditions on high risk sex and HIV/STIs. These findings highlight the importance of examining HIV risk factors through a syndemics framework [53, 54]. Understanding how co-occurring conditions together may influence HIV risk is especially important in sub-Saharan Africa, where numerous conditions have been named “syndemics” in relation to HIV/AIDS, including: alcohol abuse, IPV, food insecurity, and other co-morbidities such as tuberculosis [55–58]. Moreover, the communities most devastated by HIV are commonly burdened by a number of psychosocial conditions that tend to cluster in impoverished areas [33, 59], and together may increase one’s risk for HIV acquisition. In our sample, men with one condition and two or more conditions reported more high risk sex acts relative to their number of sex acts compared to those with no potential co-conditions. For men, experiencing two or more conditions increased risky sex more than one alone. Women experiencing two or more potentially co-occurring conditions were also at increased risk for high risk sexual behavior, and were more likely to test positive for either HIV or an STI. Among men and women, we found preliminary evidence of a synergistic effect between depression and emotional IPV on increasing high risk sex and, among men, a synergistic effect between depression and alcohol use on testing positive for HIV or an STI.

There is a growing body of literature that recognizes the importance of employing a wider lens to understand how multiple conditions may interact to influence HIV risk and acquisition; however, more research is needed with this aim, especially in sub-Saharan Africa. The evidence of the co-occurrence and synergistic effects of behavioral and psychosocial conditions (e.g., alcohol and other drug abuse, child abuse, depression, post-traumatic stress disorder) and HIV is well-established by research from developed settings [60–64]. Relevant to the conditions examined in the present study, González-Guarda [60] has proposed an evidence-based conceptual model to explain the syndemic of substance use, IPV, HIV infection, and mental health among Hispanics in the United States. While research from sub-Saharan Africa is limited, one study demonstrated the independent and additive effect of co-occurring psychosocial problems, including food insufficiency, depression, abuse experiences, problem drinking, and sexual behaviors, on HIV risk behavior among women recruited from drinking venues in South Africa [33]. Another study similarly found evidence for a synergistic effect of IPV and alcohol use on HIV infection among pregnant women in South Africa [65].

There are several limitations to report. The data were cross-sectional, therefore we cannot infer causation or the direction of the relationship between depression, IPV, or alcohol use and our HIV risk outcomes. Our data relied mainly on self-report, which is subject to recall bias, as well as social desirability. However, our findings are strengthened by the use of biological measures of HIV and STI outcomes in addition to self-reported high risk sex. Our small sample size and low prevalence of alcohol use in particular, however, may have limited our ability to detect associations between our independent variables and the HIV/STI outcome, as well as our power to test interactions between the syndemic conditions. The discrepant findings between high risk sex and HIV/STI positivity may be due in part to the small cell sizes for the HIV/STI variable. Alternatively, the discrepant findings may also be due to a general lack of concordance between behavioral and biological indicators of HIV risk. A recent paper details and explains reasons for this frequently observed lack of concordance [66].

In our sample, 20% of men reported emotional IPV and 5.6% reported physical IPV. However, it is possible that men’s reporting of IPV could be explained by retaliation violence; that is, women’s perpetration of IPV may be in self-defense or a reaction to male perpetrated violence [67]. We were unable to test whether retaliation violence could partially or fully account for men’s experience of IPV, as we did not measure perpetration of, or motivation for, violence. Finally, we recruited participants from an outpatient clinic in Uganda, limiting the generalizability of our findings to similar settings.

## Conclusions

This study demonstrates the co-occurrence of depression, IPV, and alcohol use in men, and depression and IPV in women, in an outpatient setting in rural Uganda. The co-occurrence of these factors were associated with greater HIV risk, highlighting the need for a more holistic approach to HIV prevention research and programming. Specifically, public health programming aimed to reduce HIV transmission may be more effective when addressing the multiple, intersecting behavioral and psychosocial conditions that may increase one’s risk for HIV. Increasingly, research and programming are expanding HIV prevention interventions to incorporate IPV [30, 68, 69] and alcohol and substance abuse [70–72] as core components to HIV/AIDS reduction, with promising results. However, more research and programming that simultaneously addresses depression, IPV, and alcohol use as intersecting risk factors for HIV is needed in Uganda.

## Abbreviations

AIDS: Acquired immune deficiency virus
AUDIT: Alcohol use disorders identification test
CAPI: Computer-administered personal interview
CT: *Chlamydia trachomatis*
HIV: Human immunodeficiency virus
IPV: Intimate partner violence
NG: *Neisseria gonorrhoeae*
PCR: Polymerase chain reaction
PITC: Provider-initiated HIV testing and counseling
RPR: Rapid plasma reagin
STI: Sexually transmitted infection
TPHA: *Treponema pallidum* hemagglutination
